# Memory Functioning in Children with Epilepsy: Frontal Lobe Epilepsy, Childhood Absence Epilepsy, and Benign Epilepsy with Centrotemporal Spikes

**DOI:** 10.1155/2014/218637

**Published:** 2014-08-03

**Authors:** Ana Filipa Lopes, José Paulo Monteiro, Maria José Fonseca, Conceição Robalo, Mário Rodrigues Simões

**Affiliations:** ^1^Faculty of Psychology, University of Coimbra, Apartado 6153, 3001-802 Coimbra, Portugal; ^2^Neuropaediatric Unit, Garcia de Orta Hospital, Almada, Portugal; ^3^Neuropaediatric Unit, Coimbra Paediatric Hospital, Coimbra, Portugal

## Abstract

Specific cognitive deficits have been identified in children with epilepsy irrespective of results on intelligence tests. Memory deficits are traditionally attributed to temporal lobe epilepsy, whereas the impact of frontal lobe epilepsy on memory functions has remained controversial. The aim of this study was the examination of memory abilities in other childhood common epilepsy syndromes (frontal lobe epilepsy (FLE), childhood absence epilepsy (CAE), and benign epilepsy with centrotemporal spikes (BECTS)) and the influence of epilepsy-related variables. Memory was examined in 90 children with epilepsy (each epilepsy group consisted of 30 children), aged 6–15, and compared with 30 control children. Children with FLE showed significant deficits in verbal and visual memory. In addition, type of epilepsy, earlier age at epilepsy onset, and longer active duration of epilepsy were associated with memory problems. Seizure frequency and treatment, however, did not influence memory performance. This study indicates that children with FLE show greater risk of developing memory deficits than children with CAE or BECTS, thus highlighting the importance of assessing also memory functions in frontal lobe epilepsy.

## 1. Introduction

Memory plays an important role in school performance, as school age children have to learn and integrate new information every day. Memory problems are common in patients with epilepsy and may be differently affected by the cause, course, or treatment of epilepsy. Memory deficits have been documented especially in children with temporal lobe epilepsy [1–7]. But several studies have shown that memory problems may be present in other epilepsy syndromes, such as frontal lobe epilepsy [7–12] and idiopathic generalized epilepsy including childhood absence epilepsy [7, 13, 14] and benign epilepsy with centrotemporal spikes [15–17].

The study of memory combines the characterization of the phases of encoding, storage, and retrieval of information, which has been associated with temporal as well as frontal networks. Whereas children with temporal lobe epilepsy seem to reveal most of all difficulties with recalling, those with frontal lobe epilepsy seem to be more prone to interference and show difficulties organizing the material to be learned and finding mnemonic strategies [9, 10, 18]. Children with frontal lobe epilepsy experience memory difficulties that have been attributed to primary attention problems and difficulties in applying successful encoding strategies. However this association between memory deficits and frontal lobe epilepsy is still controversial, as some authors have reported no memory difficulties [19, 20].

Although memory deficits are more often present in focal epilepsies, some studies have also found memory problems—especially in visual memory—in children with childhood absence epilepsy [10, 13, 14, 21], while others have not found significant differences in memory functioning in these epileptic syndromes [22, 23]. However these children are usually integrated in a miscellanea group that includes not only children with childhood absence epilepsy, but also juvenile absence seizures, juvenile myoclonic epilepsy, and grand mal seizures on awakening, integrating several generalized idiopathic epileptic syndromes.

There is also an extensive literature on the neuropsychological functioning of children and adolescents with benign epilepsy with centrotemporal spikes that includes the study of memory functions. Some studies with this population have identified deficits on verbal memory tasks [16, 24, 25]. Other studies found no memory deficits in this subgroup [22, 26]. In this group of children with epilepsy, memory problems seem secondary to problems on the language domain, as children with benign epilepsy with centrotemporal spikes show deficits that involve most of all verbal functioning [17, 24, 26].

Besides type of epilepsy there are multiple factors that may contribute to memory problems in children with epilepsy. These include age at epilepsy onset [7, 18, 21, 27], duration of active epilepsy [7, 18], seizure frequency [21, 28, 29], and treatment [30, 31]. However, only few studies in the paediatric population have studied the impact of these epilepsy-related variables on memory. Moreover, often the evaluation protocols do not include measures of memory.

The aim of this study was to examine memory abilities in children with common childhood epilepsy syndromes (frontal lobe epilepsy (FLE), childhood absence epilepsy (CAE), and benign epilepsy with centrotemporal spikes (BECTS)) and compare them with matched controls. We hypothesize that children with FLE will present worse performances on memory tasks, especially on verbal memory. Finally, we will examine the influence of epilepsy-related variables (type of epilepsy, age at epilepsy onset, duration of active epilepsy, frequency of seizures, and treatment) on memory functions.

## 2. Material and Methods

### 2.1. Participants

Memory was examined in 90 children with epilepsy (30 with frontal lobe epilepsy (FLE), 30 with childhood absence epilepsy (CAE), 30 with benign epilepsy with centrotemporal spikes (BECTS)), and 30 controls. Children with epilepsy were recruited from neuropaediatric units of the Hospital Garcia de Orta and Coimbra's Paediatric Hospital. This study was approved by the institutional review boards of both institutions and families and children gave their consent to participate. The healthy control children were chosen from the group that was previously used to standardize Coimbra's Neuropsychological Assessment Battery, to match the experimental group for socioeconomic level, age, and gender.

The participants with epilepsy were classified by child neurologists using the International League against Epilepsy criteria [32, 33]. All the children with FLE had performed neuroimaging testing. Children with epilepsy were selected based on the following inclusionary criteria: (i) they were between 6 and 15 years of age; (ii) they were diagnosed with FLE, CAE, or BECTS; (iii) they were administered the Wechsler Intelligence Scale for Children, third edition [34], and obtained a full scale IQ ≥ 70 [35]; and (iv) they were receiving no more than two antiepileptic medications.

### 2.2. Measures

Memory functioning was evaluated using memory tests from Coimbra's Neuropsychological Assessment Battery [36]. Coimbra's Neuropsychological Assessment Battery is a comprehensive assessment instrument, directed towards the assessment of Portuguese children's neuropsychological development and functioning. It contains a diversified group of subtests which address the domains of attention, executive functions, memory, language, and motor functions [37]. The different subtests are transformed in scaled scores with a mean of 10 and a standard deviation of 3. The following memory tests were administered.

#### 2.2.1. List Learning

This test assesses the child's ability to learn and evoke a list of words. Children must learn a list of 15 words over four consecutive learning trials (*learning*). After learning an interference list that is repeated only once, the child is asked to recall the first list of words (*immediate recall trial*). After an interval of 20 to 30 minutes, the child must recall once more the learning list (*delayed recall trial*). Finally, 45 words are presented and the child must identify which words belong to the learning list (*recognition trial*).

#### 2.2.2. Rey Complex Figure Test

This is a classic measure of visual memory, visuospatial constructional ability, and planning. Children are instructed to copy a complex geometric figure and then reproduce it from memory after three minutes (*immediate recall*) and then 30 minutes (*delayed recall*). Given the fact that this paper aims to study memory functioning, the copy results will not be described.

#### 2.2.3. Corsi Block Tapping Test

This is a test of visual working memory. Children have to remember blocks that were tapped in sequence by the examiner. Immediately after children repeat the sequence demonstrated by the examiner, these sequences are presented on a wooden board with 9 blocks numbered on the side of the examiner (children do not have visual access to the blocks numeration). At each difficulty level (starting at two blocks) two different trials are presented.

### 2.3. Statistical Analysis

Statistical analysis was performed using the Statistical Package for the Social Sciences (SPSS, Chicago, IL, USA—version 17.0). Associations between categorical variables were analysed using chi-square test. To test mean differences in demographic and clinical variables and in memory scores across the three types of epilepsy (FLE, CAE, and BECTS), analysis of variance (ANOVA) was used with post hoc analysis using* Tukey HSD*. Simple regression analysis was used to analyse the effects of epilepsy-related clinical variables (type of epilepsy, age at epilepsy onset, active duration, frequency of seizures, and treatment). Results were judged statistically significant if the *P* value was identical to or smaller than 0.05.

## 3. Results

Table 1 summarizes the demographic (age at testing, gender, and parental education) and clinical characteristics (age at epilepsy onset, seizure frequency, active duration of epilepsy, and treatment) of the participants, 90 children and adolescents with epilepsy (30 FLE; 30 CAE; 30 BECTS), and 30 controls, between the ages of 6 and 15 years old. No significant differences were found between the clinical groups and the control group for age at testing and parental education. However for the variable gender the group with FLE differed from the CAE, BECTS, and control groups. These can be explained by the fact that FLE is more frequent in male children [8, 9]. We tested for gender differences on the memory tests performed and no differences were found between boys and girls. On the neurological characteristics of the experimental samples no significant differences were observed between the groups for any of the epilepsy-related variables in analysis (age at epilepsy onset, seizure frequency, active duration of epilepsy, and treatment). The group with FLE was composed of 7 children with structural aetiology and 23 with unknown aetiology.

### 3.1. Differences between Groups

As can be seen in Table 2, significant differences were observed for the following list learning test trials: learning (*F*(3,116) = 5.039, *P* = 0.003), immediate recall (*F*(3,116) = 3.322, *P* = 0.022), and recognition (*F*(3,116) = 5.191, *P* = 0.002) and also for the Corsi block tapping test (*F*(3,116) = 3.098, *P* = 0.030). Post hoc analysis revealed a consistent pattern in which children with FLE showed the worst performance on memory testing: compared to controls, FLE performed worse on the list learning-learning trial (*P* = 0.001); on the list learning-immediate recall trial (*P* = 0.023); on the list learning-recognition trial (*P* = 0.005); and on the Corsi block tapping test (*P* = 0.017). The group with BECTS scored significantly lower than controls on the recognition trial of the list learning task (*P* = 0.004). For the Rey complex figure test (immediate and delayed recall trials) the differences between the clinical groups and the control group did not reach statistical significance.

Given the fact that 7 children from the FLE group had structural lesions, a second analysis including only the other 23 cases with unknown cause was performed. Significant differences were still found for list learning trials—learning (*F*(3,109) = 5.770, *P* = 0.001), immediate recall (*F*(3,109) = 4.434, *P* = 0.006), and recognition (*F*(3,109) = 6.779, *P* = 0.000)—and for the Corsi Block Tapping Test (*F*(3,109) = 3.057, *P* = 0.031). Post hoc analysis revealed that children with FLE performed worse than controls on the same 4 trials: on the list learning-learning trial (*P* = 0.001); on the list learning-immediate recall trial (*P* = 0.005); on the list learning-recognition trial (*P* = 0.000); and on the Corsi block tapping test (*P* = 0.017). Also on learning trial of the list learning test the group with FLE had significantly lower scores than CAE (*P* = 0.047) and BECTS (*P* = 0.017).

### 3.2. Risk Factors Related to Epilepsy

Table 3 shows the results for the linear regression analysis. Regression coefficients were not significant for frequency of seizures and treatment. Lower scores on the list learning test-learning trial (FLE versus AE: *P* = 0.042; FLE versus BECTS: *P* = 0.021) were associated with the type of epilepsy. Also lower scores on the list learning test-learning trial (*P* = 0.055) were related to a longer duration of epilepsy. Finally, lower scores on the Corsi block tapping test (*P* = 0.002) were associated with younger age at epilepsy onset.

## 4. Discussion

Mnemonic deficits are traditionally considered characteristic of temporal lobe epilepsy. However, the presence of memory problems in children with FLE is a question still open in the literature. Results of the present study confirm that children with FLE demonstrate a higher risk of developing memory problems than children with CAE or with BECTS, highlighting the importance of assessing memory functions especially in FLE. Age at epilepsy onset and duration of active epilepsy were the other epilepsy-related variables that were associated with lower memory scores.

### 4.1. Type of Epilepsy

Our study indicates that children with FLE show greater risk of developing memory problems. Memory deficits occurred mainly during the learning phase of the verbal learning task (list learning test) and the impairment on the immediate recall and recognition trials seems to be the consequence of problems during the initial encoding of information. Verche et al. [38] identified similar verbal memory deficits using the same task, where children with FLE applied encoding and recall strategies less effectively than those of control children. The authors suggest that the deficits found could be explained by the executive functioning deficits commonly detected in patients with FLE. These results appear to be consistent with those of Jambaqué et al. [10] and Lendt et al. [11], who both demonstrated memory impairment did not only occur in participants with temporal lobe epilepsy, but also in children suffering from FLE. These FLE samples showed particularly poor performances in verbal memory tasks that required planning and organizational strategies. Visual memory has been less well studied in children with FLE. Contrary to previous studies [9], we did not find deficits on visual memory, as assessed by the recall trials of Rey complex figure test. On the visual working memory test (Corsi block tapping test) results of children with FLE were lower than the control group, which suggests visuospatial working memory problems. The frontal lobes play an important role on the performance of these tasks, as several studies have demonstrated the activation of the prefrontal cortex for the performance of working memory tasks [39, 40]. Regarding aetiology of epilepsy, our FLE sample is mainly constituted by an unknown aetiology (77%). And in fact, authors argue that children with FLE rarely show macrostructural brain abnormalities [8]. The recent work of Braakman et al. [8, 41] hypothesized that neuronal injury associated with epilepsy could be expressed as a microstructural or functional abnormality, that simultaneously results in neurobehavioral comorbidities. 32 children with cryptogenic FLE underwent neuropsychological assessment and structural and functional magnetic resonance imaging and decreased functional frontal lobe connectivity was associated with cognitive impairment in FLE.

The existing literature suggests that most children with BECTS show intact memory skills [17, 22, 24, 26]. Our study only identified difficulties in the recalling phase (recognition) of the list learning task, presenting mean normal scores during learning and recall on all of the other memory tasks. Attention problems that have been previously reported in children with BECTS [17, 42–44] may justify the difficulties with recognition of items previously learned and failure to resist interference.

It is very important to consider memory problems in the context of other cognitive pathology, namely, in the domains of attention, executive functions, and language. Considering that memory deficits in children seem to be a part of a much more diffuse impairment [27, 45]. Clinically, the memory profile of each child should be analysed in the context of other neurocognitive abilities and this is only possible when conducting extensive and comprehensive neuropsychological assessment protocols.

### 4.2. Age at Epilepsy Onset and Duration of Active Epilepsy

Concerning the impact of the clinical variables related to epilepsy, besides type of epilepsy, age at epilepsy onset and duration of active epilepsy were the most significant risk factors for memory difficulties in our study. Seizure frequency and the number of antiepileptic drugs were not associated with memory functioning.

In the present study younger* age at epilepsy onset* was associated with poorer performance on the Corsi block tapping test that assesses visual working memory. The negative impact of an earlier age at epilepsy onset on cognitive functions has been largely documented, including memory functions [12, 18, 21]. In our study, an earlier age at epilepsy onset was related to a lower performance on a task that assesses visual working memory but that is also sensitive to attention and executive functions deficits. This is a common finding on other studies. Kernan et al. [21] studied a group of children with CAE and found a significant effect for age at epilepsy onset on a digit span task testing verbal working memory abilities. And Riva et al. [18] documented a significant correlation between age at epilepsy onset of epilepsy and performance on a list learning test; these children had difficulties on learning and using clusters and these impaired their scores on recall tasks. In our study earlier age at epilepsy onset only influenced results on the task that is sensitive to executive functions performance (Corsi block tapping test). Executive functions are a heterogeneous construct that includes several subdomains (such as planning, organization, working memory, inhibition, problem solving, fluency, initiative, and anticipation) that are difficult to dissociate from other constructs, such as memory, attention, or processing speed. Executive functions are subserved by the frontal lobes and these are the latest brain regions to develop, as maturation continues throughout adolescence [46]. For this reason early age at epilepsy onset is a major risk factor for difficulties on executive functions-related skills.

A longer* duration of active epilepsy* predicted memory difficulties on the learning phase of the list learning task. That is, children and adolescents with more years of active epilepsy were more likely to have problems on the acquisition and consolidation of knowledge, which will probably impact on their school results. A few studies have documented the impact of active duration of epilepsy on memory functions. Schoenfeld et al. [47] investigated a group of children with complex partial seizures and observed an association of a longer duration of active epilepsy with lower scores on visual memory. Nolan's group [7] studied memory functions in three childhood epilepsy syndromes (temporal lobe epilepsy, FLE, and CAE) and found that duration of epilepsy correlated negatively with memory function and thus was the most significant risk factor for memory problems. This was confirmed by another study that included only children with FLE: Riva et al. [18] found that performance on a list learning task (number of correct answers and short and long-term recall) correlated with the duration of the disorder.

### 4.3. Limitations

One of the limitations of our study was the fact that regarding verbal memory assessment we only included the list learning test, which is considered a representative task to assess memory (verbal memory). List learning is a task that includes repeated presentations of test material and is very susceptible to attention and executive functions problems.

It is frequently reported by parents and teachers that children with epilepsy experience memory deficits impairing their everyday life. Traditional memory tests may not capture all the real memory capabilities of children with epilepsy. Everyday memory might be influenced much more by attention problems than standard memory testing [48, 49]. Therefore future studies should include measures of everyday memory on neuropsychological assessment protocols, as well as other memory tests (story recall, paired word learning, sentence memory, visual recall, and memory for faces).

## 5. Conclusions

The present study demonstrates that children with FLE show significant deficits in verbal and visual memory. In addition, type of epilepsy, earlier age at epilepsy onset, and longer active duration of epilepsy were associated with memory problems. Our research findings underline the importance of offering early assessments, especially for children with FLE, with a longer duration and an early age at epilepsy onset, with extensive neuropsychological assessment protocols that include several measures of memory. Knowing the outcome of these groups of children with epilepsy in memory tests gives clinicians the possibility to establish adequate and timely school intervention plans to diminish the negative influence that this memory problems might have on their academic achievement.

## Figures and Tables

**Table 1:** Demographic and neurological features.

	FLE (*N* = 30)	CAE (*N* = 30)	BECTS (*N* = 30)	Control (*N* = 30)	*P* value
Age	M = 10.13 (SD = 2.73)	M = 9.93 (SD = 2.54)	M = 9.77 (SD = 2.43)	M = 10.13 (SD = 2.73)	0.937
Gender					
Boys	77% (*N* = 23)	30% (*N* = 9)	33% (*N* = 10)	50% (*N* = 15)	
Girls	23% (*N* = 7)*	70% (*N* = 21)	67% (*N* = 20)	50% (*N* = 15)	0.001
Years of education (mother)					
Up to 9th grade	17% (*N* = 5)	23% (*N* = 7)	20% (*N* = 6)	14% (*N* = 4)	
9th grade	30% (*N* = 9)	30% (*N* = 9)	47% (*N* = 14)	30% (*N* = 9)	
12th grade	30% (*N* = 9)	20% (*N* = 6)	20% (*N* = 6)	33% (*N* = 10)	
Superior	23% (*N* = 7)	27% (*N* = 8)	13% (*N* = 4)	23% (*N* = 7)	0.736
Age at onset (years)	M = 6.40 (SD = 3.10)	M = 6.83 (SD = 2.32)	M = 6.77 (SD = 2.43)		0.792
Seizure frequency					
No seizures (last 6 months)	57% (*N* = 17)	70% (*N* = 21)	60% (*N* = 18)		
<1 a month	30% (*N* = 9)	13% (*N* = 4)	37% (*N* = 11)		0.177
≥1 a month	13% (*N* = 4)	17% (*N* = 5)	3% (*N* = 1)		
Active duration (months)	M = 27.57 (SD = 36.24)	M = 22.63 (SD = 17.95)	M = 20.90 (SD = 26.44)		0.632
Treatment					
No medication	7% (*N* = 2)	13% (*N* = 4)	27% (*N* = 8)		
Monotherapy	80% (*N* = 24)	73% (*N* = 22)	73% (*N* = 22)		0.087
Duotherapy	13% (*N* = 4)	13% (*N* = 4)	—		

*Different from Control (*P* = 0.032), from CAE (*P* = 0.000), and from BECTS (*P* = 0.001).

**Table 2:** Memory tests scores.

	FLE (*N* = 30)	CAE (*N* = 30)	BECTS (*N* = 30)	Control (*N* = 30)	*F *	df	*P* value
	Mean (SD)	Mean (SD)	Mean (SD)	Mean (SD)			(ANOVA)
LIST learning							
Learning	6.33 (3.45)∗∗∗	8.13 (3.28)	8.47 (2.90)	9.39 (2.82)	5.039	3,116	0.003
Immediate recall	7.17 (3.06)∗	8.70 (3.38)	7.73 (3.08)	9.38 (2.20)	3.322	3,116	0.022
Delayed recall	7.83 (2.60)	8.90 (3.07)	8.40 (3.05)	9.39 (2.27)	1.744	3,116	0.162
Recognition	7.13 (3.79)∗∗	8.20 (3.63)	7.07 (3.85)∗∗	10.25 (2.72)	5.191	3,116	0.002
Rey complex figure test							
Immediate recall	8.33 (3.27)	9.00 (3.27)	8.87 (2.85)	9.57 (2.67)	0.757	3,116	0.521
Delayed recall	8.50 (3.26)	8.63 (3.73)	8.47 (3.09)	9.59 (2.84)	0.810	3,116	0.491
Corsi block tapping test	7.40 (3.14)∗	8.97 (3.44)	8.77 (3.27)	9.85 (2.74)	3.098	3,116	0.030

*Different from control (*P* ≤ 0.05).

∗∗Different from control (*P* ≤ 0.01).

∗∗∗Different from control (*P* ≤ 0.001).

**Table 3:** Memory tests: linear regression analysis.

Dependent variables	Independent variables included in the model															
	FLE versus CAE		FLE versus BECTS		CAE versus BECTS		Age at onset		Active duration		Frequency of seizures		Treatment		*r* ^2^ (for the full model)	*F*
	*β*	*P*	*β*	*P*	*β*	*P*	*β*	*P*	*β*	*P*	*β*	*P*	*β*	*P*		
List L	1.720	**0.042**	2.042	**0.021**	0.322	0.707	−0.100	0.481	−0.028	**0.055**	0.041	0.936	0.159	0.836	0.125	1.972
List IR	1.389	0.096	0.245	0.776	−1.145	0.178	−0.038	0.788	−0.015	0.291	−0.530	0.296	−0.494	0.517	0.086	1.309
List DR	1.012	0.189	0.442	0.579	−0.570	0.468	−0.088	0.496	−0.016	0.233	−0.057	0.903	−0.132	0.852	0.047	0.678
List R	0.903	0.359	−0.345	0.736	−1.248	0.216	0.068	0.681	−0.021	0.225	−0.125	0.835	−0.296	0.743	0.057	0.832
Rey IR	0.667	0.448	0.778	0.395	0.110	0.902	0.152	0.309	0.000	0.980	0.069	0.898	0.865	0.286	0.037	0.538
Rey DR	0.071	0.936	0.103	0.910	0.033	0.971	0.232	0.121	−0.003	0.840	0.059	0.912	0.703	0.386	0.046	0.663
Corsi	1.407	0.090	1.261	0.142	−0.146	0.863	0.435	**0.002**	0.020	0.170	−0.718	0.156	0.059	0.938	0.157	2.584

LIST L, list learning-learning; LIST IR, list learning immediate recall; LIST DR, list learning-delayed recall; LIST R, list learning-recognition; Rey IR, rey complex figure test-immediate recall; Rey DR, rey complex figure test-delayed recall; Corsi Corsi block tapping test.
